# Supplementary material for: A robust nitridation technique for fabrication of disordered superconducting TiN thin films featuring phase slip events
Source: Sci Rep. 2021 Apr 12;11:7888. doi: 10.1038/s41598-021-86819-6 (PMC8042045; doi:10.1038/s41598-021-86819-6)
Supplement: Supplementary file 1 — Supplementary Information [file 41598_2021_86819_MOESM1_ESM.pdf]

Supplementary Information

A Robust nitridation technique for fabrication of disordered superconducting TiN thin films featuring phase slip events

*Sachin Yadav[1, 2], Vinay Kaushik[3], M.P. Saravanan[3], R. P. Aloysius[1, 2], V. Ganesan[3] and Sangeeta Sahoo[1, 2,]**

[1]*Academy of Scientific and Innovative Research (AcSIR), AcSIR Headquarters CSIR-HRDC Campus, Ghaziabad, Uttar Pradesh, 201002, India.*

[2]*Electrical & Electronics Metrology Division, National Physical Laboratory, Council of Scientific and Industrial Research, Dr. K. S Krishnan Road, New Delhi-110012, India.*

[3]*Low Temperature Laboratory, UGC-DAE Consortium for Scientific Research, University Campus, Khandwa Road, Indore- 452001, India*

**Correspondences should be addressed to S. S. (Email: sahoos@nplindia.org)*

Contents:

1. Scanning electron microscopy (SEM) images for selected samples
2. Atomic force microscopy (AFM) images in 2D and 3D representation
3. Surface roughness from AFM images and its dependence on the annealing temperature (T_a) and on the thickness
4. Calculation of Ginzburg- Landau (GL) coherence length (ξ_{GL}) for TiN samples prepared under different growth conditions
5. IVCs of TiN samples showing both sweeps (up & down)
6. Table S1: Comparison of critical temperature (T_c) values for TiN reported in literature with the T_c obtained in the present work

1. Scanning electron microscopy (SEM) images for selected samples

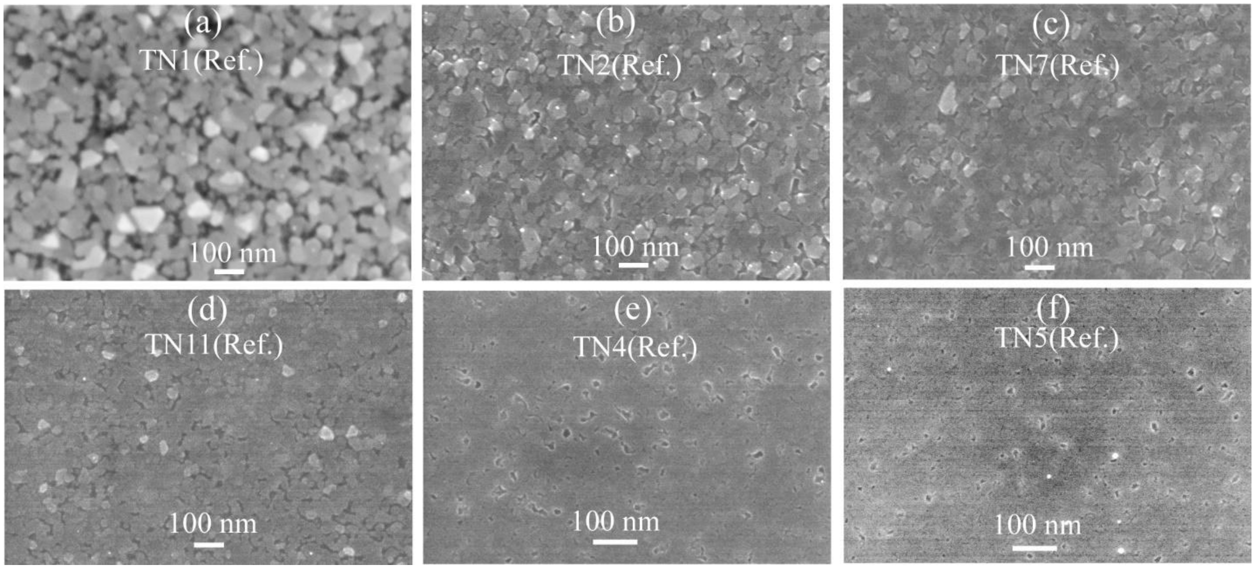

Fig. S1: *Scanning electron microscopy (SEM) images for some of the selected devices. Here, the representative samples are the reference devices that were prepared at the same run/batch of the respective measured devices. The images represent the surface morphology of the samples and the granular nature is evident. The grain size strongly depends on the annealing temperature* T_a *and the thickness.*

Scanning electron microscopy (SEM) imaging has been used to investigate the surface morphology of the measured devices and the corresponding SEM images are shown in Fig. S1. Here, we have selected identical devices that were prepared at the same run/batch of the respective measured devices and each reference sample was placed at the closest vicinity of the related measured device. For example, the sample TN1(Ref.) is the sample prepared along with the measured sample TN1 and the two samples were placed next to each other. The annealing temperature T_a and the thickness values (t) for the samples are mentioned in Table 1 in the main manuscript. Here, the relatively thicker samples with varying T_a are presented in Fig. S1(a)-(d), whereas, the two thinner samples with same T_a have been presented in (e)-(f). The images

suggest that the samples are granular in nature. Grains are bigger in size and they are clearly visible in the SEM images for the thicker sample. However, with changing thickness from 20 nm [Fig. S1(a)] to 12 nm [Fig. S1(b)], we observe that the surface roughness decreases and the surface looks smoother for the thinner sample. The surface gets further smoothed for reduced annealing temperature but with same thickness as it is clear in Fig. S1(b)-(e), for T_a 820°C, 780°C and 750°C, respectively. The average grain size for these relatively thicker samples is about 40-50 nm. Here, the grains are clearly visible in SEM images for the thicker samples, TN1, TN2, TN7 and TN11. But the grains are not clear and there is a drastic change in the surface morphology for the samples TN4 & TN5 with thickness ~ 4 nm and 3 nm, respectively. Here, the thickness is playing a crucial role and a totally different surface morphology, containing very small grains that are not clearly resolved under SEM, is obtained. In order to resolve the grains for the thinner samples, the sample topography is further studied by using atomic force microscopy (AFM) imaging which is followed in the next section.

2. **Atomic force microscopy (AFM) images in 2D and 3D representation**

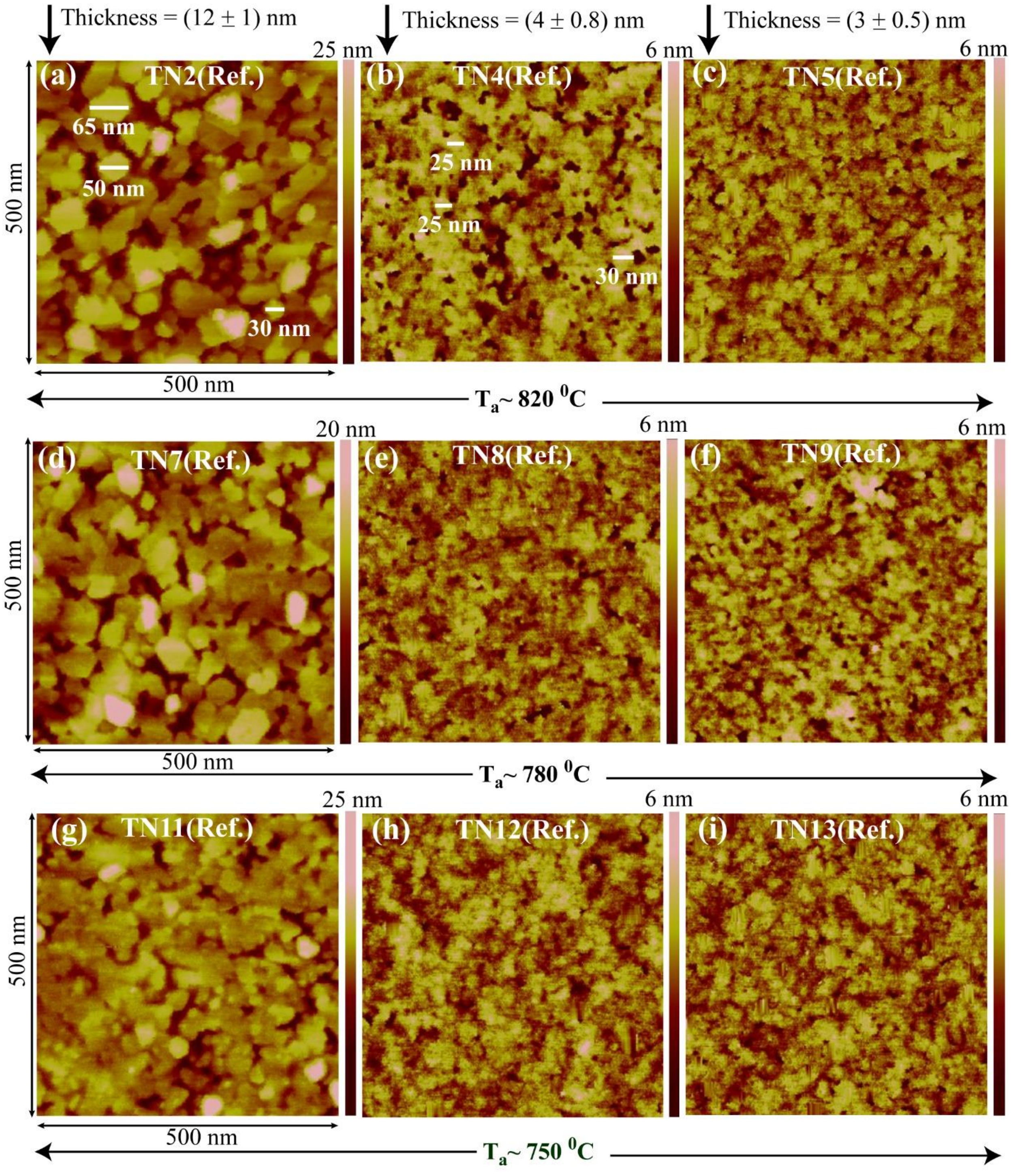

Fig. S2: *Atomic Force microscopy (AFM) images for the reference samples corresponding to all the devices presenting I-V measurements in the main manuscript. Each row represents a particular* T_a *and each column represents a particular thickness.* T_a *decreases from top to bottom whereas, thickness decreases from left to right. For having an idea about the grain size, we have marked few grains for thicker and thinner samples in (a) and (b), respectively.*

In order to have better understanding about the surface morphology, surface roughness and grain size, we have carried out the topographic study by using atomic force microscopy (AFM) imaging. In Fig. S2, we have displayed a set of AFM images for all the samples that were characterized by IVCs in the main manuscript. The top row, middle row and the bottom row correspond to T_a as 820°C, 780°C and 750°C, respectively. On the other hand, the first, second and third columns from the left represent the thickness category of 12 nm, 4 nm and 3 nm, respectively. Here, as already explained in the method section that the samples are categorized with a particular thickness which is determined by the optimized rate and from the thickness measurements obtained from various samples prepared with same deposition time. The error bars represent the range within which the measured thickness varied. As we have seen from the SEM images presented in Fig. S1, the thicker samples in first column of Fig. S2 display clear granular morphology with relatively large grain size. There is merely any difference in grain size for the samples TN2 (T_a = 820°C) and TN7 (T_a = 780°C), whereas, the surface looks smoother with smaller grain size for TN11 (T_a = 750°C). For an estimate of the grain size variation, we have marked few grains with lateral dimension for TN2 in Fig. S2 (a). A large variation of about 30-65 nm in the grain size is observed for the sample in Fig. S2 (a). Here, it should be noted that the grain size for thicker samples is about 45-50 nm as estimated from the line width in X-ray diffraction pattern [Ref. #18 in the main manuscript: *Appl. Surf. Sci*. **541**, 148465 (2021)] which is in good agreement with the values obtained from the SEM images. Contrary to the thicker samples, the middle and right columns, presenting the thinner samples, show totally different type of surface topography containing patchy type of structures of about 25-30 nm in lateral dimension as shown by the marked patches in Fig S2(b). All the samples in these two columns look almost similar and only the surface coverage in the middle columns appears to be bit more

than that of the right column. It should be noted that the thickness difference is about 1 nm between these two columns and obviously, it is very hard to find them different from each other.

Granular superconductors can be considered as an array of Josephson junctions where superconducting grains act as the superconducting islands and the grain boundaries serve as the weak -links and the macroscopic superconductivity is established by superconducting proximity effect (PE). By lowering temperature, the individual superconducting grains become superconducting locally when the temperature reaches to their transition temperature (T_C). Further the superconducting grains couple to each other by superconducting PE through the grain boundaries and the progressive coupling through PE establishes the global superconductivity in the granular matrix by establishing a macroscopic coherence. However, the establishment of macroscopic coherence depends strongly on the grain size for a particular material and the inter-granular distance. For example, when the superconducting grains are larger than the superconducting coherence length and they are closely spaced (the intergranular distance is much shorter than the respective coherence length), the global superconductivity is established within a short span of temperature and the metal-superconductor transition in $R(T)$ characteristics appears sharp. But, when the superconducting grains become comparable to characteristic length scales of electronic confinement and superconducting coherence length, finite size effects and superconducting fluctuation play a crucial role to control the metal-superconductor transition and the consequences are broad transition and incomplete superconductivity as the superconducting proximity is going to be impaired by the order parameter fluctuations. As the surface morphology, studied by SEM and AFM, suggests the variations in the grain size with sample thickness, the granularity and the grain size play a crucial role to modulate the transport properties.

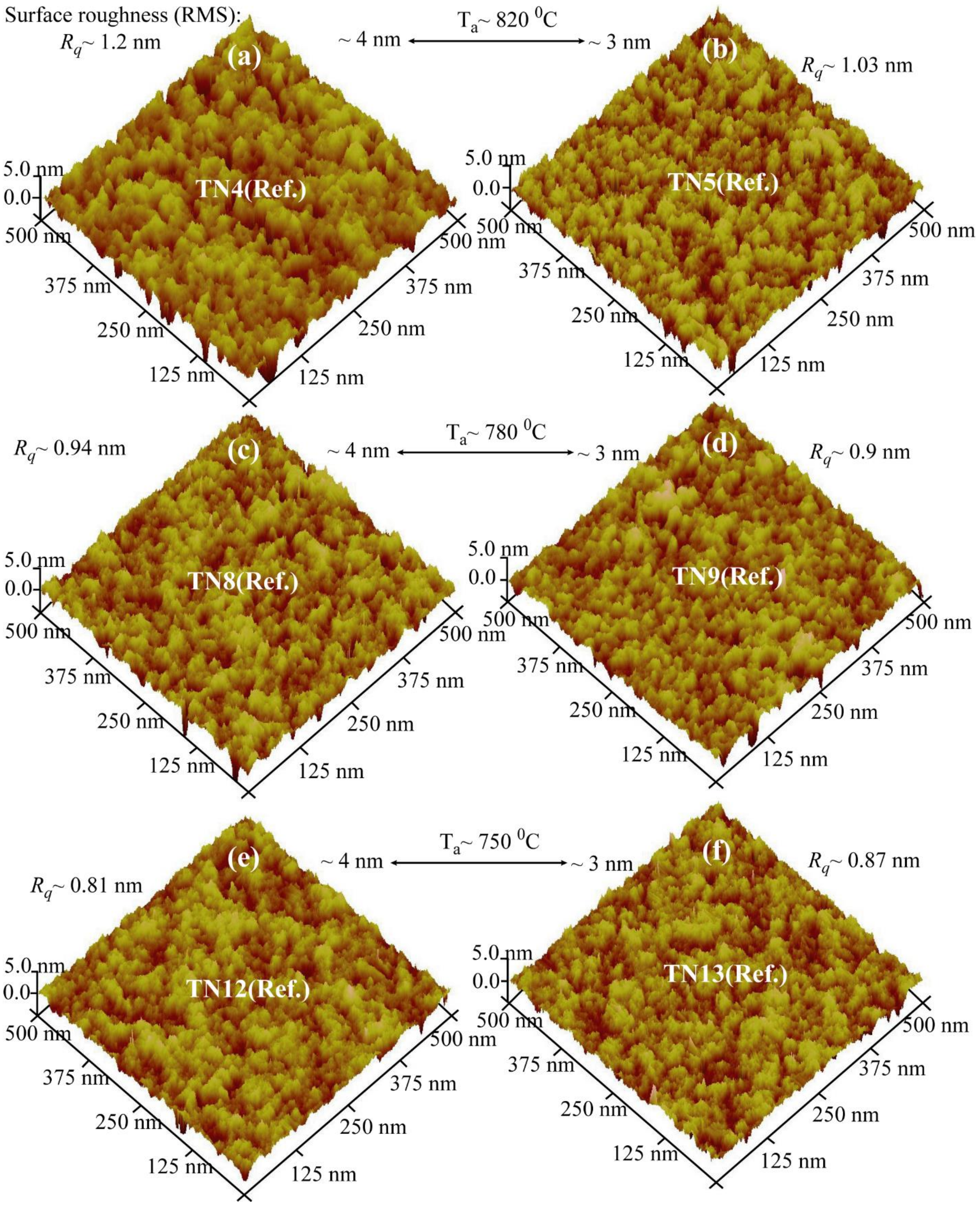

Fig. S3: *AFM images in three-dimensional (3D) representation for the thinner samples that were shown in the middle and right columns in Fig S2.*

In order to understand the constituents of those patchy structures appearing in the thinner samples, we have displayed the AFM images in 3-dimensional (3D) representation in Fig. S3. The 3D AFM images clearly demonstrate that the patchy structures are consisting of fine grains joined together. These grains are very small in dimension and to resolve them in AFM is also very difficult. However, the granular nature along with their coverage is evident from Fig. S3. The surface roughness, which is the R.M.S roughness obtained from the images for each sample, is mentioned in the respective figures.

3. Surface roughness from AFM images and its dependence on annealing temperature (T_a) and thickness

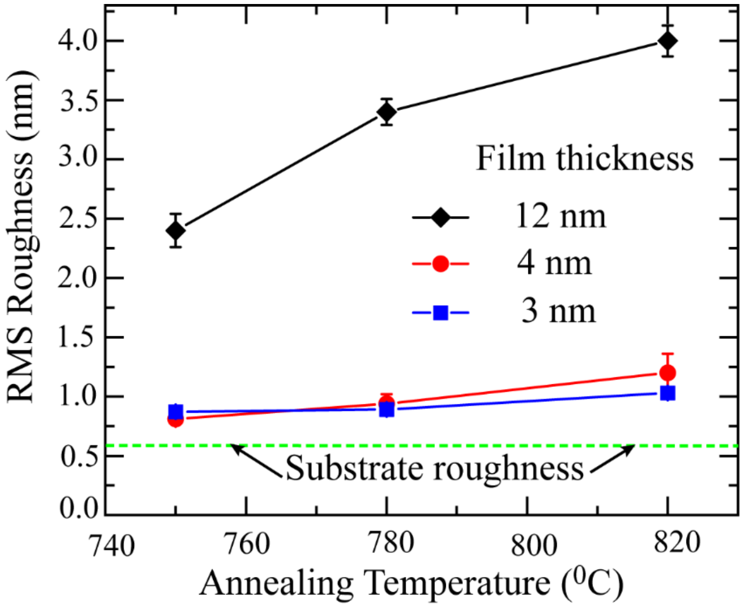

Fig. S4: *Dependence of RMS surface roughness, obtained from the AFM images, on the annealing temperature (Ta) and on the film thickness. Here, the representative three thickness categories have been selected for three selected annealing temperatures, 820℃,780℃ and 750℃. The green dashed horizontal line represents the surface roughness for the substrate.*

In Fig. S4, we have plotted the RMS surface roughness values for all the samples with respect to the annealing temperature. Here, the roughness values are taken as the average of three AFM scans for individual samples and the error bars represent the range of the variation. First of all, we see that the roughness values are much higher for the samples of thickness category 12 nm. This was evident from both SEM and AFM images presented in Fig. S2 and Fig. S3. Further with increasing T_a, roughness increases which might explain the increment in the normal state resistance with increasing T_a as presented in Fig. 1 in the main manuscript. The substrate roughness is shown by the green dashed horizontal line which is very close to the surface roughness values for the thinner samples as compared to that of the thicker ones. Hence, the effect of the substrate roughness is important for the thinner samples whereas that can be neglected for the thicker samples.

4. **Calculation of Ginzburg- Landau (GL) coherence length (ξ_{GL}) for TiN samples prepared under different growth conditions.**

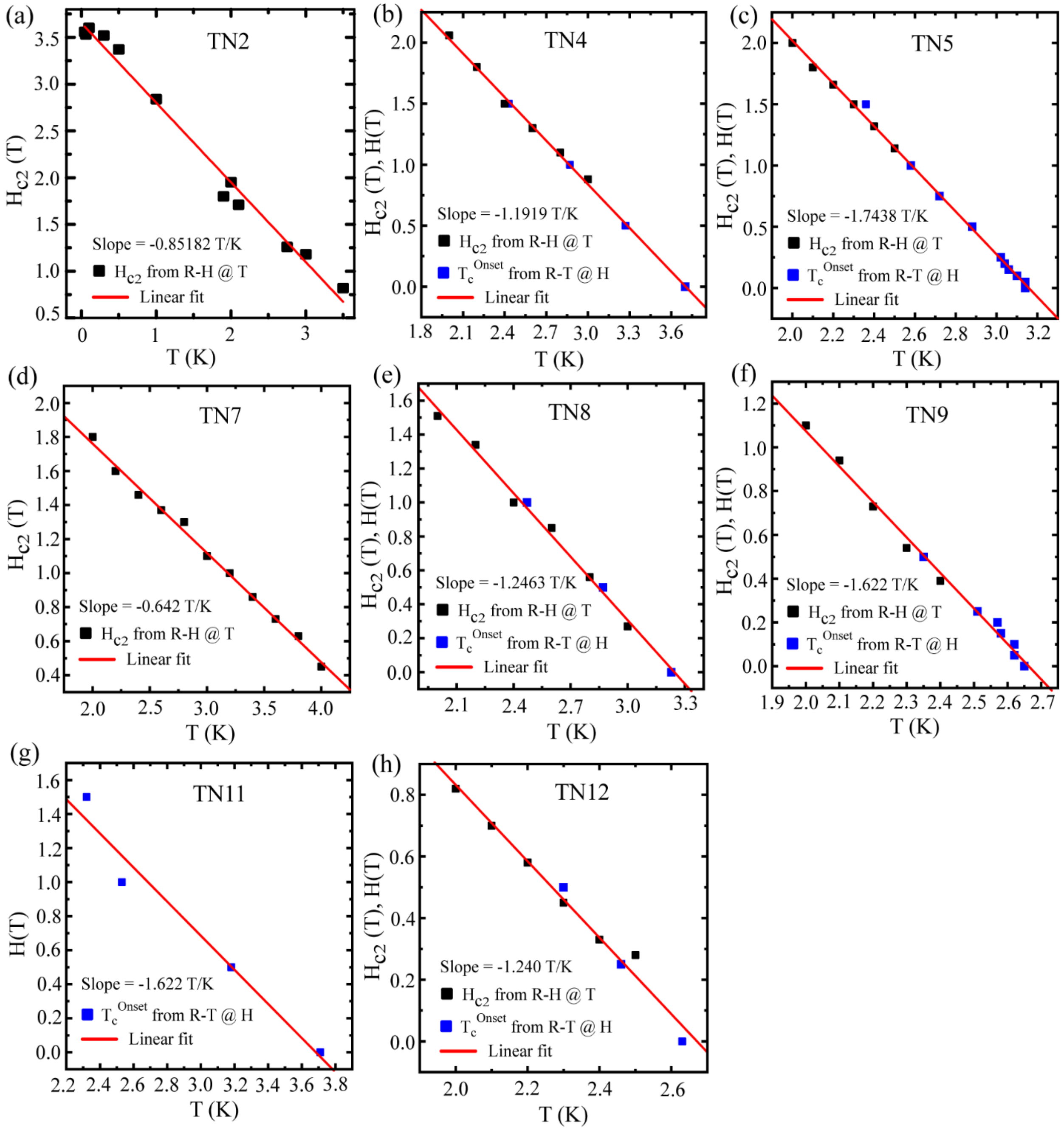

Fig. S5: *B-T phase diagram for the TiN samples. Black and blue squares are the data points collected from isothermal magnetoresistance measurements [R-H@T] and temperature dependent resistance measurements under applied field [R-T@H], respectively. Solid red lines represent the linear fits performed on the experimental data points and provide the slope for calculating the GL coherence length* ξ_{GL}.

We have calculated the Ginzburg-Landau (GL) coherence length $\xi_{GL}(0)$, by using the standard formula, $\xi_{GL}(0) = \left[\frac{\phi_0}{2\pi T_c \left|\frac{dH_{c2}}{dT}\right|_{T_c}}\right]^{1/2}$, where ϕ_0 is the flux quantum. The experimental data points for the samples TN2, TN7 &TN11 are the values of the upper critical field (H_{c2}) taken from magnetoresistance isotherms, whereas for sample TN11, experimental data points are the T_c^{Onset} values taken from the field dependent *R(T)*. However, for rest of the samples, the experimental data points are taken from both *R(T)* & *R(H)* as shown with the help of black & blue squares in Fig. S5. The extracted values from *R(T)* & *R(H)* are fitted linearly in Fig. S5 as shown by the red line. The slopes obtained from the linear fits were used for calculating the coherence length $\xi_{GL}(0)$ for all TiN samples fabricated under different growth conditions and the corresponding coherence lengths are 9.5 nm (TN2), 8.7 nm (TN4), 7.94 nm (TN5), 10.8 nm (TN7), 9.2 nm (TN8), 9.0 nm (TN9), 9.6 nm (TN11) & 10 nm (TN12), respectively.

5. IVCs of TiN samples showing both sweeps (up and down)

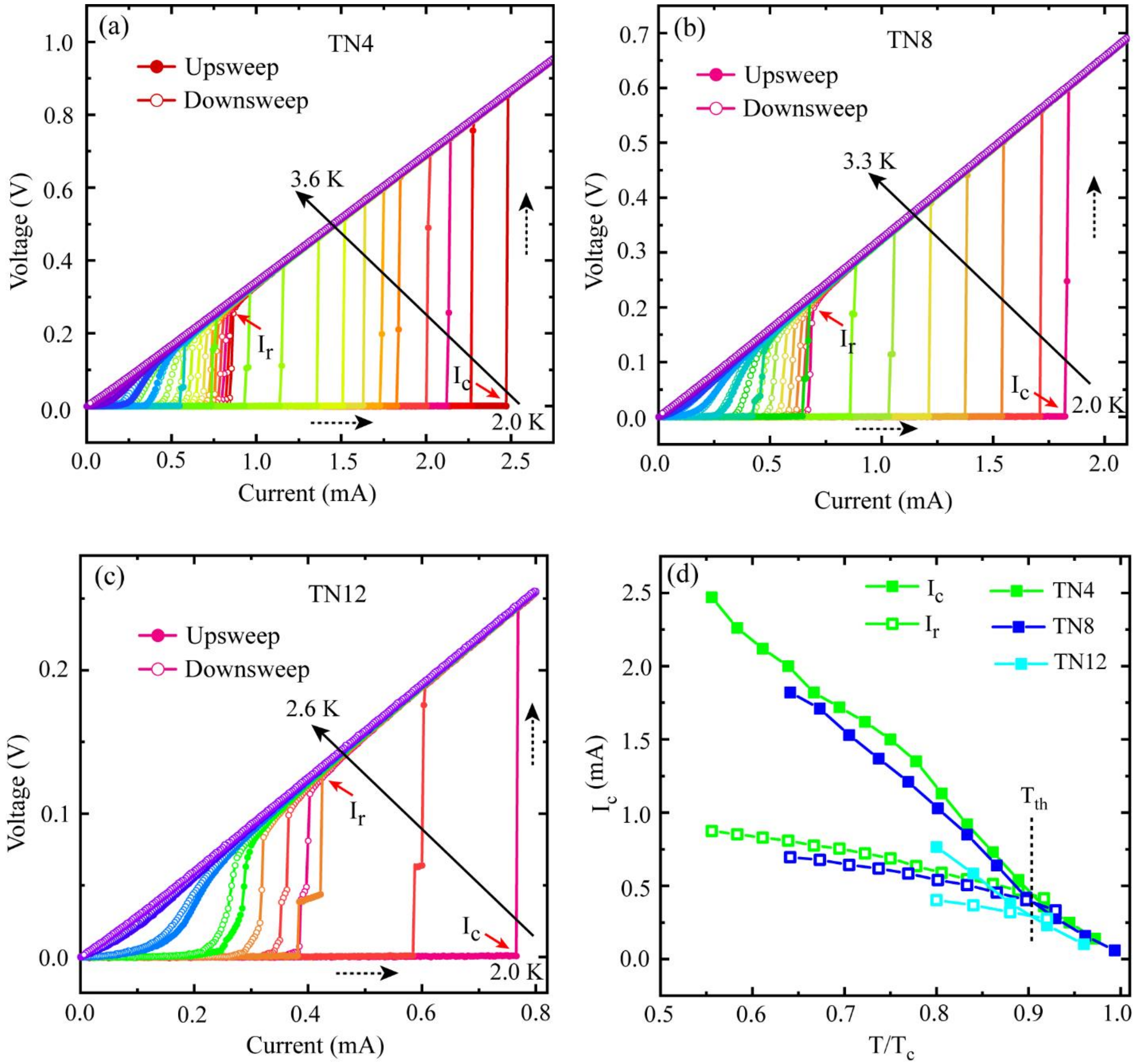

Fig. S6: *Current voltage characteristics (IVCs) of TiN thin film samples showing up and down current sweeping directions. IVC isotherms for (a) sample TN4 annealed at 820 ℃, (b) sample TN8 annealed at 780 ℃ & (c) TN12 annealed at 750 ℃. (d) The dependence of critical current (I_c) & retrapping current (I_r) on reduced temperature (T/T_c). All the three samples are having the same film thickness which is about 4 nm. Solid and empty circles in the IVC isotherms represent the up and down current sweeping directions, respectively. Current sweeping direction for upsweep is shown by black dotted arrows.*

In Fig. S6, we have presented the current voltage characteristics (IVCs) of TiN thin film samples annealed at different growth temperature but having the same film thickness of about 4 nm. At lower temperature, the IVCs show single step sharp transition from superconducting to metallic state. The current at which the transition occurs is known as the critical current (I_c), whereas, the current corresponding to the onset of superconducting state from the normal state is defined as retrapping current (I_r). I_c and I_r are marked by the solid red arrows in Fig. S6(a). Moreover, we do see similar kind of single step transition in *IVCs* for sample TN8 as shown in Fig. S6(b). However, multiple resistive steps appear in the transition from superconducting to metallic state for the sample TN12 as shown in Fig. S6(c). Further, as these two characteristic currents (I_c & I_r) are different, the IVCs appear to be hysteretic with respect to the current sweeping direction. With increasing temperature, the hysteresis gets reduced and vanishes at T_C. Hysteretic IVCs are commonly observed in granular superconducting films due to Joule heating effect, which locally increases the effective temperature and hence reduces the critical current. The dependence of I_c and I_r on temperature is shown in Fig. S6(d). Here, at lower temperature (far away from T_c), hysteresis is more prominent and it reduces as the temperature approaches close to the T_c. However, at a particular temperature, I_r becomes equal to I_c and the temperature is known as the threshold temperature (T_{th}) which is marked in Fig. S6(d) with a vertical dotted line and above this temperature I_r becomes higher than I_c.

6. Table S1: Comparison of critical temperature (T_c) values for TiN reported in literature with the T_c obtained in the present work

**Reference*	T_C (K)	*Thickness, (nm)*	**Growth technique**	*Substrate*	*Crystallographic Structure*
Ref #21	4.6	80	Reactive sputtering	Sapphire	
	3.6	22			
	2.6	15			
Ref #23	4.5	35	Reactive sputtering	Si (100)	Poly-crystalline
Ref #24	0.7 K≤T_C≤4.5K	20 nm≤ t ≤100 nm	Reactive sputtering	Si (100)	Poly-crystalline
Ref #25	4 K<T_C <4.5 K (T_C is determined by 90% resistance criteria)	62 nm	DC biased sputtering	HF-cleaned Si (100)	Poly-crystalline; with dominant (200) orientation
Ref #29	6.0	Size: 1x1x1mm^3	Chemical-vapor Deposition		Single crystal
Ref #30	5.25	40 nm	Plasma enhanced MBE	MgO (001)	
Ref #22	5.4	~ 200 nm	Reactive sputtering	HF-cleaned Si (100)	Single crystal
Ref #26	4.5 K	100-200 nm	Reactive sputtering	HF-cleaned Si (001)	Poly-crystalline
Ref #27	3.0 K	8.9 nm	ALD	Si (111)	Preferentially (200) oriented
	4.6 K	109 nm			
Ref #28	3.4 K	18 nm	Plasma-enhanced ALD	Si (110)	
Ref #31	4.83 K	~ 100 nm	PLD	MgO, SiN, Sapphire	Poly-crystalline
Current work	***3.0 K≤T_C≤4.8 K***	***3 nm ≤ t ≤20 nm***	***Substrate mediated nitridation***	***SiN***	***Poly-crystalline***

**** References are mentioned in the main manuscript***

In Table S1, we have presented a list of references that represent the best achieved critical temperature (T_c) related to superconductor-metal phase transition for TiN. The last row of the table contains the results from the current work. Along with the T_c values, we have also collected the film thickness, adopted techniques and the crystallographic structure. First of all, we find that our results are comparable and compared to many of the reported results, they offer even better T_c, particularly if we pay attention to the thickness range. However, higher T_c values are reported in References 23, 25 and 24. The highest reported T_C for this material is obtained from Ref #23 which actually dealt with bulk single crystal of size 1x1x1mm^3 prepared by CVD technique. It is obvious that the single crystalline materials are expected to offer a better T_C compared to poly-crystalline films. In Ref # 23 & 24, the material is single crystalline. Further, the growth technique is very important for the quality of the materials. For example, MBE is known for high quality epitaxial thin film growth as appeared in Ref # 25 with higher T_C for this material grown on MgO with nearly same lattice parameter.

For polycrystalline TiN films, usually grown by conventional reactive magnetron sputtering, the current work presents the most promising results as far as the superconducting critical temperature is considered. Here, we should note that all the reported works, presented in the table corresponding to T_c values higher than 4 K, are obtained from much thicker films than the present work. Even though above a certain thickness, T_c is supposed to be independent of thickness but we do observe variations in T_c values with thickness (e.g. References 17, 29, 20 and in the current work) but in our case T_c becomes independent of thickness at 15nm and above.
